# Complete Heart Block Induced Torsades de Pointes

**DOI:** 10.7759/cureus.55169

**Published:** 2024-02-28

**Authors:** Benjamin A Szumowski, Youstina Mary Beshay-Taylor, Ramy Sadek, Nader Attia

**Affiliations:** 1 Internal Medicine, Southwest Healthcare Medical Education Consortium, Temecula, USA; 2 Cardiology, Southwest Healthcare Medical Education Consortium, Temecula, USA

**Keywords:** r-on-t phenomenon, prolonged qt interval, high-degree atrioventricular block, torsades de pointes, polymorphic ventricular tachycardia

## Abstract

High-degree atrioventricular node block is a known cause of bradycardia. Heart rate and QT interval have an inverse relation. Therefore, bradycardia can lead to prolonged QT interval, which can predispose patients to Torsades de Pointes, a life-threatening arrhythmia. Correcting the underlying etiology can often reverse the arrhythmia and prevent recurrence. For this reason, recognizing the etiology of this arrhythmia plays an essential role in management.

## Introduction

Torsades de Pointes (TdP) is a type of polymorphic ventricular tachycardia (PMVT) that is associated with QT interval prolongation. TdP can be identified by its typical appearance of the QRS complex changing in amplitude and morphology in a twisting pattern around an isoelectric line [1]. TdP is important to be able to recognize as it is a life-threatening arrhythmia requiring prompt diagnosis and treatment. The mortality of TdP is approximately 10-20% [2]. The etiologies for TdP include ischemia, genetic components, electrolyte derangements, drugs, and less commonly, bradycardia [3]. A thorough history is essential when considering the etiology of TdP and should include components such as alcohol consumption and family history of sudden cardiac death. Understanding the underlying etiology guides the treatment. Herein, we present a unique case of a patient with bradycardia secondary to a high-degree atrioventricular block complicated by TdP requiring emergent permanent pacing.

## Case presentation

**Figure 1:**
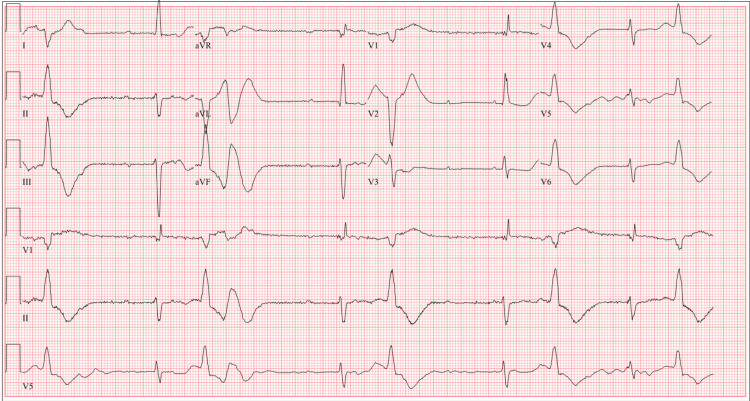
EKG showing complete heart block with prolonged QT segment and frequent premature ventricular complexes EKG: Electrocardiogram

**Figure 2:**
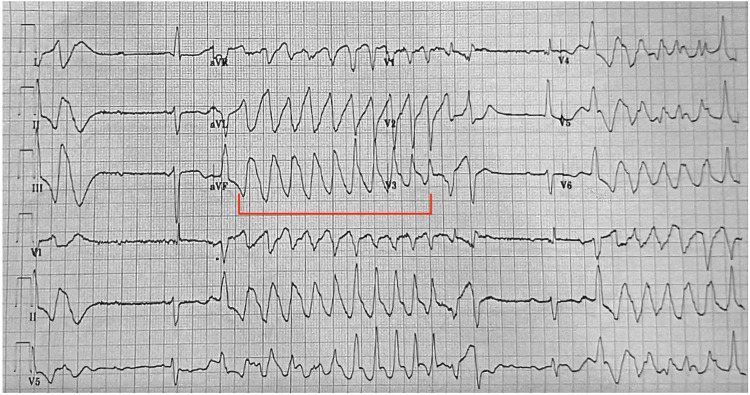
EKG showing complete heart block with intermittent non-sustained PMVT Non-sustained PMVT highlighted with red bracket EKG: Electrocardiogram; PMVT: Polymorphic ventricular tachycardia

Here we present a 75-year-old female with a past medical history of atrial fibrillation on metoprolol and dabigatran, heart failure with preserved ejection fraction, hypertension on amlodipine, chronic obstructive pulmonary disease, obstructive sleep apnea, and diabetes who presented with subacute shortness of breath for one week and witnessed syncopal episode. The syncopal episode was described as becoming dizzy while ambulating in her home and losing consciousness but she denied any trauma. On presentation, vital signs were notable for a heart rate of 48 beats per minute, blood pressure of 130/70 mmHg, and normal oxygen saturation on room air. The patient’s physical exam was largely unremarkable except for bradycardia on auscultation of the heart, but otherwise, no murmurs were appreciated. The patient's other home medications included aspirin, lisinopril, albuterol, fluticasone-salmeterol, metformin, bupropion, atorvastatin, and sertraline. Social history was negative for alcohol consumption and family history showed no sudden cardiac death. Laboratory testing notable for normal high-sensitivity troponin, B-type natriuretic peptide, and thyroid function tests. The patient's chemistry panel including potassium was within normal limits except for a magnesium level of 1.7 mg/dL and creatinine of 1.61 mg/dL. Complete blood count showed hemoglobin of 9.1 gm/dL. Chest X-ray revealed a mildly enlarged cardiac silhouette but no pleural effusions or pulmonary congestion. The echocardiogram showed a normal global systolic left ventricle function and moderately dilated bilateral atria. Electrocardiogram (EKG) showed bradycardia with complete heart block, prolonged QT interval of 634 milliseconds, frequent premature ventricular contractions, and frequent non-sustained PMVT (Figures 1, 2). Upon chart review, the patient had prolonged QT interval noted up to 570 milliseconds in the previous admission several years prior. Initially, emergency department physicians did not recognize PMVT and erroneously managed this as monomorphic ventricular tachycardia (MVT). Therefore, the patient was started on amiodarone with a loading infusion of 150 mg consistent with MVT management recommendations. Shortly after starting amiodarone, it was noted that the patient became more bradycardic. Cardiology was emergently consulted. EKGs were reviewed and noted to be consistent with PMVT, likely induced by bradycardia secondary to high-grade atrioventricular block. Amiodarone was immediately discontinued. Given the concern for bradycardia-induced TdP, the patient received intravenous magnesium sulfate and was emergently transcutaneously paced at a heart rate of 60 beats per minute with the resolution of her arrhythmia. The patient was briefly monitored in the ED until she was safely transferred to the intensive care unit for close monitoring before permanent pacemaker placement. The electrophysiologist evaluated the patient and she emergently underwent permanent pacemaker placement on the same day. The patient was then placed in the ICU for continued management and close monitoring given high illness acuity. Laboratory testing did not yield electrolyte abnormalities that would be contributory to the patient’s occurrence of TdP. Further workup with an echocardiogram demonstrated normal biventricular function and no significant valvular disease. The patient unfortunately did not undergo cardiac angiography as the hospital is not a cardiac center and cardiac catheterization was unavailable. During the hospitalization, the patient was monitored. She remained asymptomatic. Continuous telemetry monitoring did not reveal additional events of TdP. It was concluded that the TdP observed on initial presentation was most likely secondary to the high-grade atrioventricular block-induced bradycardia. Therefore, the reversal of bradycardia through the placement of a pacemaker resulted in the resolution of the arrhythmia.

## Discussion

TdP is a type of PMVT that is associated with a prolonged QT interval. This prolongation of the repolarization phase increases the risk of early after-depolarization (EAD) events. When an EAD is timed during the preceding T wave, this is termed an R-on-T phenomenon and potentiates the risk of developing TdP [3]. The mechanisms for QT prolongation lending itself to TdP include congenital, drug-induced, electrolyte derangements, and less commonly, bradycardia [1,3]. Bradycardia prolongs the repolarization phase through multiple mechanisms including a decrease of outward current by sodium-potassium pumps at a slower heart rate, more complete decay of the delayed rectifier potassium current, and the inhibition of reverse-use-dependent potassium channels [4]. These mechanisms can be inhibited through an increase in the heart rate. Drugs that are typically used for ventricular tachycardia, such as amiodarone, would be contraindicated. In particular, this patient was about to be started on an amiodarone drip after the loading infusion by the emergency department just prior to cardiology’s involvement. Amiodarone has been documented to prolong QT and cause drug-induced TdP. Interestingly, IV administration, in particular, of amiodarone and the female sex increases this risk of TdP [5]. Therefore, our case demonstrates the importance of prompt identification of TdP and evaluation for reversible underlying etiologies.

## Conclusions

TdP is a life-threatening arrhythmia that is associated with prolongation of the QT segment and the repolarization phase. It should be promptly recognized and treated. Identifying the underlying etiology is crucial in guiding the management. Bradycardia and high-degree atrioventricular blocks can prolong the repolarization phase and increase the risk for TdP. Treatment of bradycardia through cardiac pacing can minimize the risk of recurrent TdP.
